# P-452. C-Reactive Protein and Procalcitonin Levels During Bloodstream Infections in the Pediatric Cardiac Intensive Care Unit at Duke University Hospital

**DOI:** 10.1093/ofid/ofaf695.667

**Published:** 2026-01-11

**Authors:** Mayse Nasser, Jillian H Hurst, Michael J Smith

**Affiliations:** Duke University Hospital, Durham, NC; Duke University Hospital, Durham, NC; Duke University, Durham, North Carolina

## Abstract

**Background:**

Postoperative infections remain a significant concern in pediatric patients following cardiac surgery. While inflammatory biomarkers such as procalcitonin (PCT) and C-reactive protein (CRP) may help with early detection of infection, their effectiveness in distinguishing infection from postoperative inflammation remains uncertain.

**Methods:**

We conducted a retrospective analysis of pediatric patients (< 18 years) admitted to the PCICU after cardiac surgery between January 1, 2022, and December 31, 2024. We extracted and evaluated PCT and CRP levels obtained on the same day as the cultures. Positive blood cultures were defined using the National Healthcare Safety Network (NHSN) standardized definition, with single cultures for coagulase-negative Staphylococcus (CoNS) excluded. We calculated the median and interquartile ranges (IQRs) for CRP and PCT both overall and by organism. We used the Wilcoxon rank-sum test to compare CRP and PCT between positive and negative blood cultures. All analyses were performed using Stata, version 18.5.

**Table 1:**
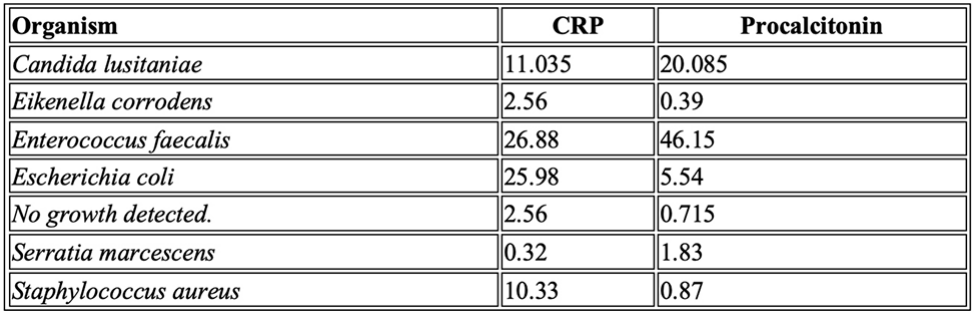
PCT and CRP by organism.

**Results:**

We identified 659 patients (731 admissions); the median age was 1.5 months, and 55% were male. During the study period, 150 patients had 459 blood cultures; of these, 29 samples from 21 patients were positive. Median procalcitonin (PCT) levels were higher in positive cultures [8.1 (IQR 0.87-29.7)] compared to negative ones [0.7 (IQR 0.23–2.08)]. Similarly, median C-reactive protein (CRP) was elevated in positive cultures [13.19 ( IQR 5.12-26.88)] versus negatives [IQR 2.59 (0.86-7.02)]. CRP and PCT values varied by organism with *Enterococcus faecalis* exhibiting the highest levels for both markers (Table 1). Both markers were significantly elevated in patients with positive cultures (p < 0.001).

**Conclusion:**

Our findings indicate CRP and PCT are significantly elevated in PCICU patients with blood stream infection as compared to those with negative blood cultures. Additional studies will be needed to assess CRP and PCT in the context of other common infection sites.

**Disclosures:**

Michael J. Smith, M.D., M.S.C.E, Pfizer: Grant/Research Support

